# Supplementary material for: The causal relationship between the human gut microbiota and pyogenic arthritis: a Mendelian randomization study
Source: Front Cell Infect Microbiol. 2024 Nov 26;14:1452480. doi: 10.3389/fcimb.2024.1452480 (PMC11629706; doi:10.3389/fcimb.2024.1452480)
Supplement: Supplementary file 1 [file DataSheet1.zip › mendelian test/ebi-a-GCST90027689.csv_leave_one_out.pdf]

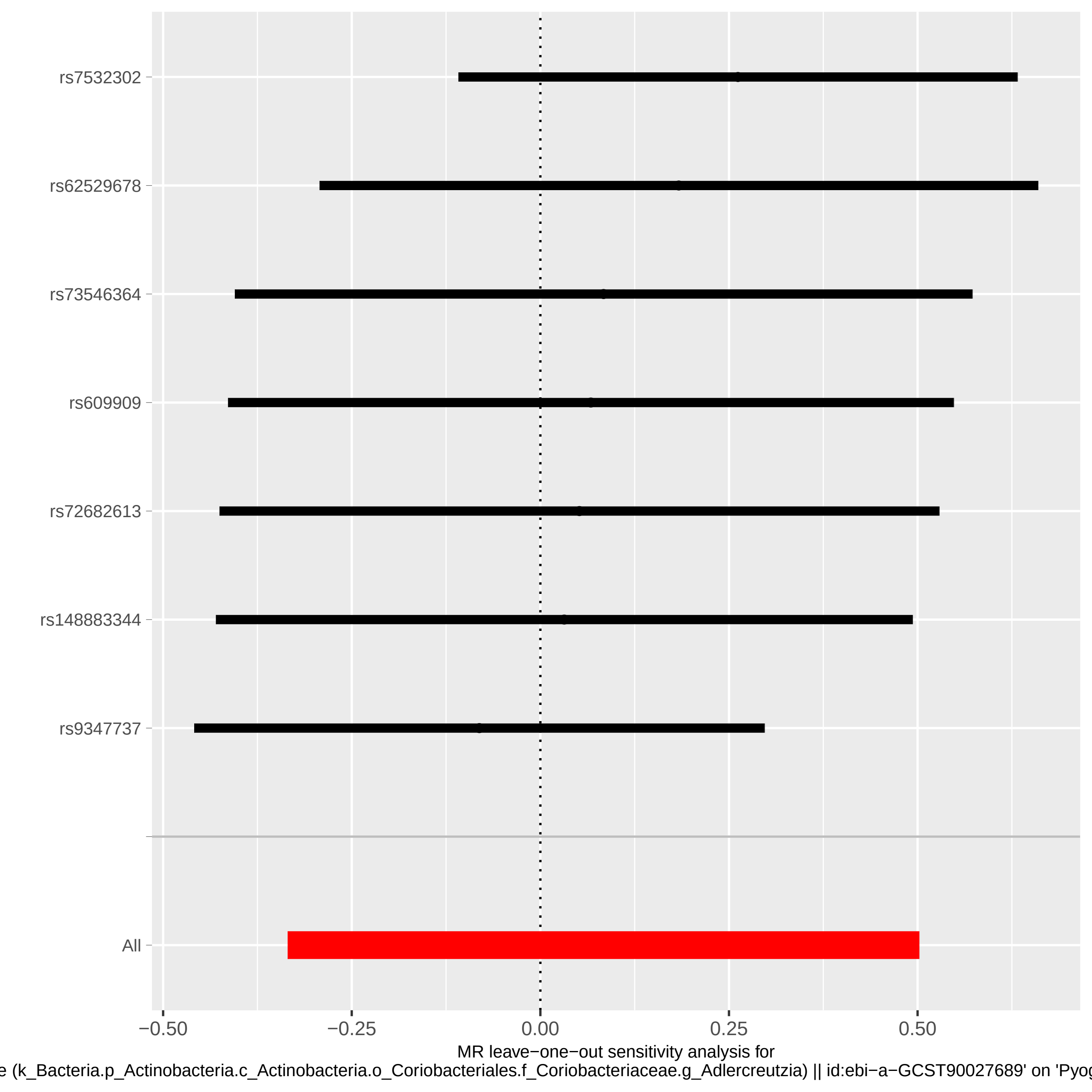
rs7532302
rs62529678
rs73546364
rs609909
rs72682613
rs148883344
rs9347737
All
−0.50
−0.25
0.00
0.25
0.50
MR leave−one−out sensitivity analysis for
e (k_Bacteria.p_Actinobacteria.c_Actinobacteria.o_Coriobacteriales.f_Coriobacteriaceae.g_Adlercreutzia) || id:ebi−a−GCST90027689' on 'Pyog
